# Effect of Clinically Relevant CAD/CAM Zirconia Polishing on Gingival Fibroblast Proliferation and Focal Adhesions

**DOI:** 10.3390/ma10121358

**Published:** 2017-11-27

**Authors:** Nicholas G. Fischer, Jeffrey Wong, Andrew Baruth, D. Roselyn Cerutis

**Affiliations:** 1 Department of Physics, College of Arts and Sciences, Creighton University, 2500 California Plaza, Omaha, NE 68178, USA; Fisc0456@umn.edu (N.G.F.); JeffreyWong@creighton.edu (J.W.); 2 Department of Oral Biology, School of Dentistry, Creighton University, 2802 Webster Street, Omaha, NE 68178, USA

**Keywords:** dental materials, dental implant, mucosal seal, zirconia, gingival fibroblast, abutment

## Abstract

Mucosal seal formation around dental abutments is critical to the successful integration of dental implants into the human oral cavity. No information exists for how clinically relevant polishing procedures for computer-aided design and computer-aided manufactured (CAD/CAM) zirconia abutments affects cellular responses important to mucosal seal formation. CAD/CAM zirconia was divided into four groups for clinically relevant polishing utilizing commercial polishing heads: control, coarse, coarse plus medium, and coarse plus medium plus fine. Surfaces were analyzed with scanning electron microscopy (SEM), atomic force microscopy (AFM), and optical profilometry (OP). Subsequently, human gingival fibroblasts (HGFs) were seeded onto the zirconia surfaces. Proliferation was measured via a quantitative SEM technique and focal adhesion kinase (FAK) phosphorylation status was measured by an enzyme-linked immunosorbent assay (ELISA). Results showed an increase in proliferation on all polished surfaces as compared to the control. Phosphorylation of FAK at tyrosine 397 (Y397) was up-modulated on the control surfaces. The associated cell adaptation is discussed. In all cases, FAK phosphorylation was greater at 24 h than 48 h. These results suggest that clinicians should be mindful of the effects of abutment polishing methodology, as this may have an impact on early mucosal seal formation.

## 1. Introduction

The regeneration of the soft tissue surrounding dental implants is important in the prevention of bacterial infections and peri-implant diseases, which are some of the leading causes of implant failure [1]. The transmucosal portion of the implant, the abutment, forms a mucosal seal with the gingival tissue, protecting it against bacterial challenges, achieving pleasing aesthetics, and contributing to a host of factors implicated in implant success [2,3,4]. Human gingival fibroblasts (HGFs) are one of the predominant cell types composing the gingival tissue of the mucosal seal [5,6]. HGF proliferation is key for mucosal seal formation [7]. However, as this is influenced by the contact guidance and anchorage dependence of HGFs, the substratum influences proliferation [8,9]. Adhesion of HGFs to abutments is important to a robust mucosal seal and is largely dictated by focal adhesions (FAs) [10]. Focal adhesion kinase (FAK), a key factor to the formation of FAs, has been shown to be modulated based on the substratum [11,12,13,14]. Therefore, the material substratum selection of dental abutments is critical to the formation of effective mucosal seals.

The surfaces of abutments are well studied for their effect on cellular behavior. Biologically-inspired surface functionalization [15,16] and surface energy [17,18] approaches have all been tested in vitro to elucidate positive cellular responses implicated in mucosal seal formation. Similarly, a wide range of work has examined the effects of surface topography on cellular responses important to mucosal seal formation, including proliferation and adhesion [7,8,19,20,21]. Zirconia (Zr) has been introduced as a material for implant abutments due to its biocompatibility [22], tooth-like color [23], low plaque retention [4], and bulk mechanical characteristics [24]. Titanium, the “gold standard” of abutment materials, is well-studied but its blueish-gray color is a concern to patients [25]. Therefore, Zr abutments have gained popularity, particularly in computer-aided design/computer-aided machining (CAD/CAM) dentistry.

CAD/CAM is one of the fastest growing technologies in dentistry and was first developed in the 1980s in response to advancing technologies, time efficiency, and environmental concerns [26,27]. As the field has matured, CAD/CAM Zr implant abutments are expected to increase in usage [28]. Due to variability in computer-aided machining, Zr processed via CAD/CAM, including abutments, requires individual clinician finishing and polishing to achieve final, correct anatomy [29,30]. These processes affect the topography of the surface at all size scales, including those relevant to cellular response. While the polishing of Zr has been studied, including rotational frequency [31], application force [32,33], and application time [34], these surface modification strategies have not been applied to abutments with a specific focus on cellular response. In fact, an extensive literature search revealed no prior work examining the effects of clinically relevant abutment polishing procedures on cellular responses for Zr surfaces. While a great deal of work has examined the role of topography on the cellular responses implicated in mucosal seals, none of that work has been undertaken with CAD/CAM Zr surfaces prepared in a clinically relevant manner, i.e., polished with polishing heads. Therefore, in many situations, surfaces that would be atypical for clinical settings are being studied instead. While previous work has examined sand paper polished [13,21,35], sand-blasted [3,19], and laser-ablated [12] surfaces for their effects on HGF responses, no work exists for surfaces prepared with any sort of polishing head, which is the current recommendation by many manufacturers [30]. In addition, Irving et al. [36] recently commented that “to date (2017) the use of abrasive polishing methods has been largely overlooked for developing textured surfaces for manipulating cell behaviour.” In short, clinically relevant polishing of CAD/CAM Zr has not been studied for its implication on HGF adhesion and proliferation, and thus for mucosal seal formation and implant success.

The present research evaluated the response of HGFs to clinically relevant Zr surfaces polished with clinically available polishing heads. The exhaustive study used scanning electron microscopy (SEM) for quantifying HGF proliferation and a FAK enzyme-linked immunosorbent assay (ELISA) for phosphorylation status of FAK to assess critical focal adhesions. This combined a cellular and molecular approach. Further, the resultant polished surfaces were evaluated with SEM, optical profilometry (OP), and atomic force microscopy (AFM), which allowed for quantitative analysis of surface topography for a wide bandwidth of lateral length resolutions.

## 2. Materials and Methods

### 2.1. Specimen Fabrication

As-received (ø = 95 mm, 15 mm thick) monolithic Zr (ZirkonZahn, Gais, Italy) pucks were nominally sectioned into 2.5 mm thick plates with a low-speed diamond wet-saw (Model C, Pistorius Machine Co., Hicksville, NY, USA), flat ground with a silicon carbide sanding paper (220-grit, 3M, Saint Paul, MN, USA), and then machined into disks (ø = 5 mm, 2.5 mm thick) with a vertical milling unit (TRAK K2 SX, Southwestern Industries, Rancho Dominguez, CA, USA). Water from machining was removed by placing disks in a drying oven at 78 °C (Precision 658 Compact Oven, Thermo Fisher, Waltham, MA, USA) for at least 72 h. Samples were sintered according to manufacturer specifications for ramp rates and holds in a box furnace with a final hold temperature of 1480 °C for 2 h (Lindberg/Blue M 1700 °C Tube Furnace, Thermo Fisher, Waltham, MA, USA) with the associated control unit (Lindberg/Blue M CC59246PCOMC-1, Thermo Fisher, Waltham, MA, USA). Following sintering, disks were polished with 320-grit silicon carbide sanding paper (Norton, Worchester, WA, USA) until a thickness of 2.0 mm was uniformly achieved (final dimensions of ø = 5 mm, 2.0 mm thick). 

### 2.2. Specimen Polishing and Finishing

Specimens were randomly divided into four study groups:

1 Control: 320-grit sanded;
2 Coarse (C): Finishing (ZilMaster Coarse (green), Bullet Shape, Shofu Dental Corp., Kyoto, Japan);
3 Coarse plus Medium (C+M): Finishing (as previously described for Group C) and polishing (ZilMaster Medium (blue), Bullet Shape, Shofu Dental Corp., Kyoto, Japan);
4 Coarse plus Medium plus Fine (C+M+F): Finishing and polishing (both as previously described for Group C+M) and final polishing (ZilMaster Fine (yellow), Bullet Shape, Shofu Dental Corp., Kyoto, Japan).

A rotary tool (Dremel 4000, Racine, WI, USA) was mounted in a mounting unit (Dremel 220, Racine, WI, USA) and set for polishing at 15,000 RPM, as recommended by the manufacturer and previous work [31]. Following our recent work on polishing force and the resultant topography of Zr [33], polishing was done by hand for 15 s, at an application force of approximately 3 N [29], with the Zr surface perpendicular to the polishing head at one stroke per second. A stroboscope (Strobotac Type 1531-A, General Radio Co., Boston, MA, USA) verified the rotation frequency.

### 2.3. Optical Profilometry, Atomic Force Microscopy, and Scanning Electron Microscopy

Following polishing, specimens were sonicated in ethanol for 10 min to clear any polishing debris before imaging. OP measurements were performed on a Proscan 2100 noncontact optical profilometer (Scantron Industrial Products, Ltd., Taunton, UK). Associated Proscan software was used for analysis. A cut-off length of 0.25 mm, sampling length of 0.30 mm × 3.0 mm, and operational scan rate 300 Hz, following ISO 4288 recommendations, were utilized [37]. A range of 0.02 μm to 0.1 μm in *R*_a_ can be measured with these specifications for non-periodic surface profiles. Ten disks were imaged per group.

AFM measurements were performed in an acoustic and mechanical noise isolation chamber on an Agilent 5420 AFM (Agilent, Santa Clara, CA, USA) in ambient conditions (22 ± 2 °C and 40 ± 20% relative humidity) conditions. Micrographs were obtained in a constant force contact mode with a Silicon Nitride cantilever (spring constant of 0.2 N/m and a tip radius of ≤10 nm), (BudgetSensors, Sofia, Bulgaria) at 512 lines per image at a 3.92 Hz scan rate. Micrographs (30 μm × 30 μm) were analyzed with Gwyddion (Central European Institute of Technology, Brno, Czech Republic) and Agilent’s PicoView software (Agilent, Santa Clara, CA, USA). Ten disks per group were imaged in three areas near the center of the polished region, as determined with optical microscopy. Roughness was quantified in terms of *R*_a_, the arithmetic average (over all line scans in each image) of the absolute values of the profile height deviations from the mean line. Finally, the control and three groups of polished Zr were imaged with a TM3000 Tabletop Scanning Electron Microscope (Hitachi High-Technologies Corporation, Tokyo, Japan) using an accelerating voltage of 15.0 kV at a magnification of ×1800 and ×200. 

### 2.4. Cell Culture

HGFs were isolated from healthy gingival tissue as previously described [38]. The HGFs were grown in Dulbecco’s Modified Eagle Medium (Corning Cellgro, Manassas, VA, USA) supplemented with 10% fetal bovine serum (Gibco, Grand Island, NY, USA) and 0.002% antibiotic (Primocin, Invivogen, San Diego, CA, USA); this will be referred to as “complete media”. The cells were grown at 37 °C in a humidified 5% carbon dioxide atmosphere. Confluent cells were subcultured by 0.25% trypsin/EDTA (ethylenediaminetetraacetic acid) trypsinization. Medium was replaced every three days. All experiments were performed using cells up to passage 12, following the methodology of Könönen et al. [7].

### 2.5. Cell Seeding

Before seeding, the Zr disks were washed and then sonicated (20 min) with 1% Liquinox (Alconox, Inc., White Plains, NY, USA) to remove any debris or contaminants, rinsed extensively with water and then de-ionized water, dried, and then dry-heat disinfected for 1 h at 170 °C. Previous research has shown routine autoclave treatment does not phase transform dental Zr [39]. Based on preliminary results, 2 × 10^4^ cells/well in complete media were seeded onto Zr surfaces in 96 well-plates. Briefly, using special low-adhesion, low-retention pipette tips, 10 μL of cell suspension was pipetted onto each disk and incubated for 90 min, similar to Zheng et al. [18]. Following this, complete medium was added (140 μL/well) and well-plates were further incubated for 24 or 48 h. Following cell seeding and incubation (24 h and 48 h), cells were fixed by immersion in 95% ethanol for 5 min and air-dried, then imaged at a magnification of ×200. Cellular proliferation was determined by imaging three independent 0.25 mm^2^ areas (approximately upper, center, and lower) per disk (n = 12 disks), as previously described [40]. Cell counting was performed by two separate examiners (N.G.F. and J.W.).

### 2.6. FAK ELISA

Prior to running the ELISA assays, cell viability on the zirconia disks was confirmed by crystal violet staining, essentially as described by Nagatomo et al. [41] and Franken et al. [42]. The observed classical fibroblast morphology under optical microscopy, along with the staining, confirmed cell viability on the non-toxic Zr surface across all specimens. A commercially available focal adhesion kinase (FAK) enzyme linked immunosorbent assay (ELISA) (FAK (Phospho) [pY397] Human ELISA Kit, Thermo Fisher, Waltham, MA, USA) was utilized to determine the phosphorylation status of FAK. Following incubation, seeded disks were washed twice in ice cold Hank’s balanced salts solution (HBSS) (HyClone, Thermo Fisher, Waltham, MA, USA) and fixed in Theralin (Grace Bio-Labs, Bend, OR, USA) for 30 min. Theralin was developed to provide optimal preservation of phosphorylated proteins (versus traditional formalin fixation, which has been reported to exhibit low or variable preservation of phosphorylation status, depending on the protein [43]). Following fixation, disks were washed twice in ice-cold HBSS and lysed on ice for 30 min with a cell extraction buffer (including 2 mM sodium orthovanadate and 1% Triton X-100; Invitrogen, Carlsbad, CA, USA) supplemented with a protease inhibitor cocktail (Halt, Thermo Fisher, Waltham, MA, USA). The extracted solution was added to the provided ELISA capture plates coated with detection antibody (for FAK pY397) and the plates were incubated for 3 h. Following this and washing, the anti-rabbit immunoglobulin G horseradish peroxidase (IgG-HRP) was added and incubated for 30 min. Next, chromogen (TMB) was added and incubated for 30 min. The reaction was stopped with the stop solution (0.16% sulfuric acid) and the plates read immediately at 450 nm on a microplate reader (Synergy H1, BioTek, Winooski, VT, USA). Seven disks per group (based on a power analysis at 75% power), performed in triplicate, were separately analyzed at 24 h and 48 h of incubation. Results are presented as optical density (O.D.).

### 2.7. Data Analyses

Mean *R*_a_ values, cell counts, and optical density measurements were compared with a one-way analysis of variance (ANOVA) followed by Tukey’s HSD (honest significant difference) post hoc test. GraphPad Prism 7.0c (GraphPad Software, San Diego, CA, USA) was utilized for calculations. A *p* value of <0.05 was considered statistically significant. Standard deviation was included when appropriate.

## 3. Results

### 3.1. Zr Microscale Topography, SEM and OP

**Figure 1:**
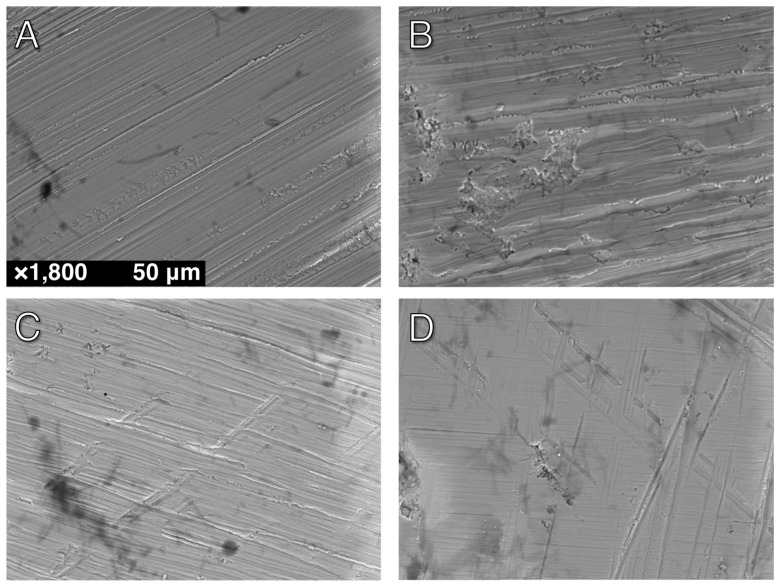
Representative scanning electron micrographs of Zr surfaces at ×1800: (**A**) Control; (**B**) Group C (Course); (**C**) Group C+M (Course+Medium); (**D**) Group C+M+F (Course+Medium+Fine). See text for details on group definitions.

**Figure 2:**
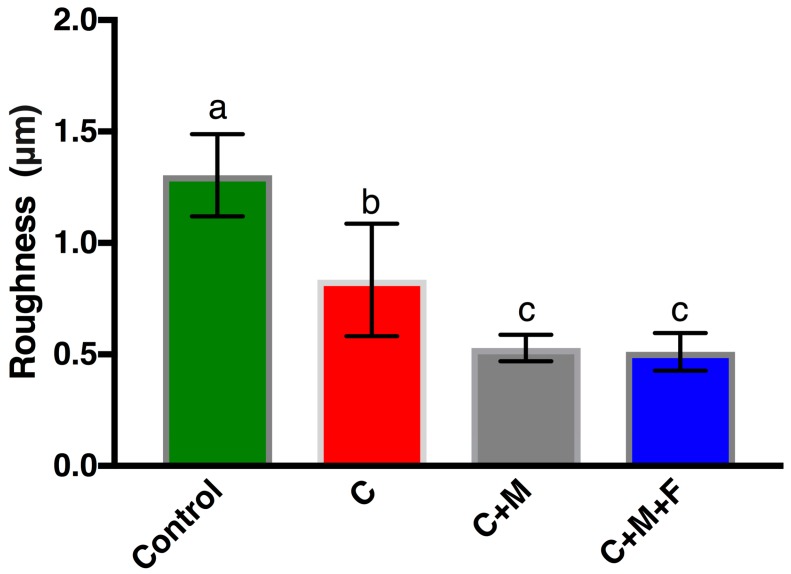
Optical profilometry-based measurement of *R*_a_ of control Zr surfaces and those following the C, C+M and C+M+F polishing steps. Bars indicate standard deviation. Same letter indicates no significant difference (*p* > 0.05).

SEM micrographs at ×1800 are shown in Figure 1 for four representative surfaces: Control, C, C+M and C+M+F. The control revealed a polished surface with clear periodic grooves across the surface, as expected following our preparation steps. Qualitatively, C polishing revealed a clear removal of this periodic surface, which was replaced with nominally flatter, wider, and sparser polishing marks. This trend continued with C+M, exhibiting a decrease in the quantity and absolute height of valleys and ridges. Finally, C+M+F maintained the overall topography of previous polishing steps with a further decrease in heights of features and the introduction of nearly imperceptible fine, shallow grooves. Reassuringly, OP *R*_a_ values, shown in Figure 2, quantitatively verified with statistical significance this qualitative behavior in surface modification. The ANOVA analysis revealed a statistically higher *R*_a_ (*p* < 0.05) for the control over all other groups, followed by C with a statistically higher *R*_a_ (*p* < 0.05) than C+M and C+M+F, with no statistical differences (*p* > 0.05) between C+M and C+M+F, where the similarities in *R*_a_ for C+M and C+M+F revealed how shallow the groove structure became following M polishing, with F making only slight further changes. The associated increase in standard deviation between C+M and C+M+F highlighted the growing disparity between larger grooves that were never fully removed and the continued polishing of finer grooves (as clearly seen in Figure 1C compared with 1D). Clearly, by this stage, *R*_a_ was dominated by the larger grooves, which were not significantly altered by adding the F polishing step.

### 3.2. Zr Nanoscale Topography, AFM

**Figure 3:**
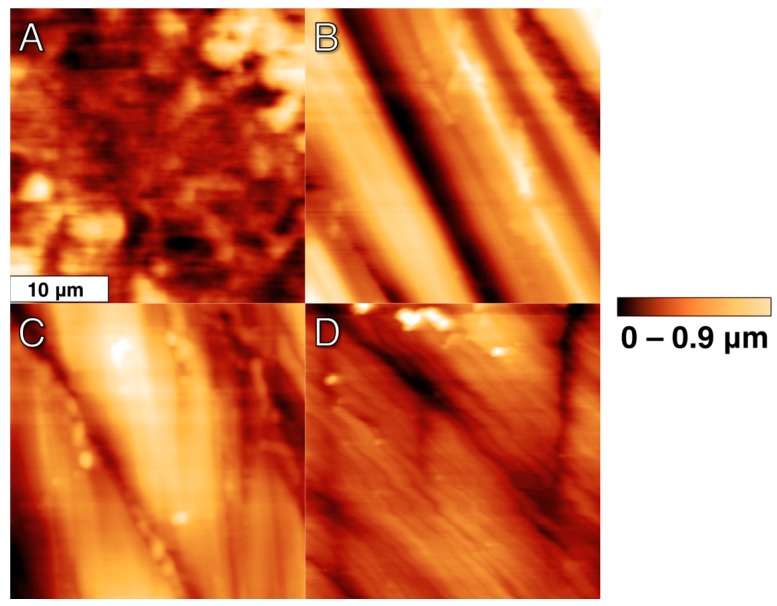
Representative plan-view topographic atomic force micrographs (30 μm × 30 μm) of Zr surfaces. False color represents height from 0 (red/dark) to 0.9 μm (yellow/light): (**A**) Control; (**B**) Group C; (**C**) Group C+M; (**D**) Group C+M+F.

**Figure 4:**
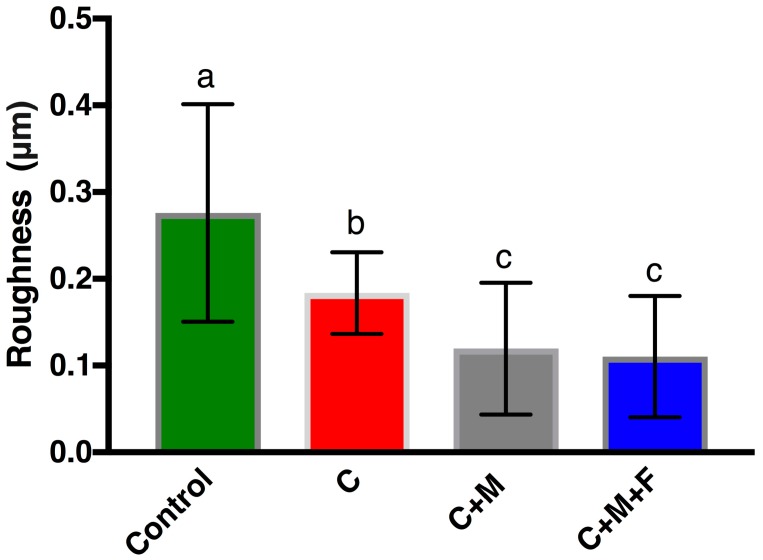
Atomic force microscopy-based measurement of *R*_a_ of control Zr surfaces and those following C, C+M and C+M+F polishing steps. Bars indicate standard deviation. Same letter indicates no significant difference (*p* > 0.05).

Four representative AFM micrographs (30 μm × 30 μm) are shown in Figure 3 with consistent false color height scales up to 0.9 μm. The addition of the height dimension helped to quantify topographies seen with SEM, and AFM provided superior lateral resolution. As expected, the nanoscale topography revealed a similar qualitative surface to the microscale techniques discussed above with some additional details. The control showed numerous, aperiodic (on this scale) pits and grooves, which are hinted at with SEM micrographs and fully detailed with AFM. C polishing effectively removed this aperiodic topography and replaced it with more periodic grooves that retain a similar depth to the control but are less frequent and flatter. Continued polishing with C+M did not change the overall topography from C, but did minimize the height deviations between the valleys and ridges. Furthermore, C+M+F further reduced the height deviations of these grooves but introduced some finer groove structures (1.47 ± 0.45 μm width) that overlay the former grooves (4.91 ± 0.70 um width). As anticipated, AFM *R*_a_ values, shown in Figure 4, quantitatively verified, with statistical significance, this qualitative behavior in surface modification. The ANOVA analysis revealed a statistically higher *R*_a_ (*p* < 0.05) for the control over all other groups, followed by C with a statistically higher *R*_a_ (*p* < 0.05) than C+M and C+M+F, with no statistical differences (*p* > 0.05) between C+M and C+M+F, where the similarities in C+M and C+M+F revealed an exchange from deeper, less periodic grooves to more shallow, more frequent grooves, as clearly seen in Figure 3C,D.

### 3.3. FAK ELISA

**Figure 5:**
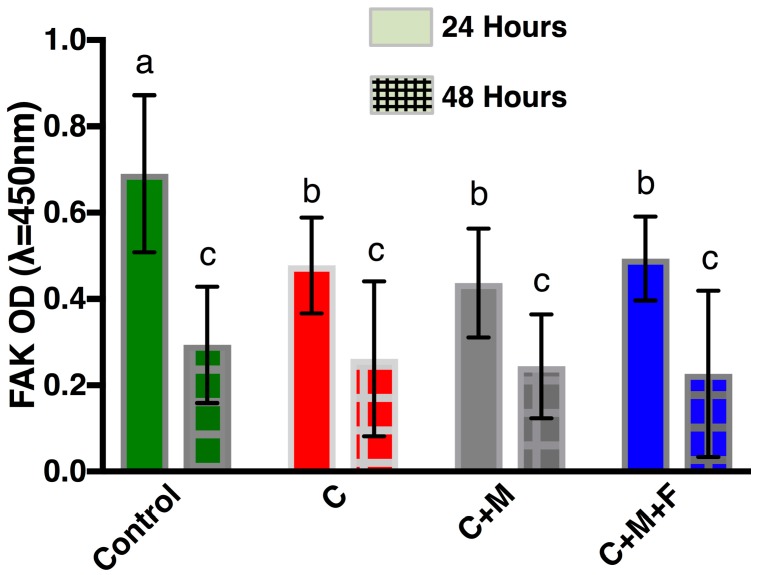
FAK phosphorylation of human gingival fibroblasts present on Zr following 24 h (solid color) and 48 h (cross-hatched) as measured by optical density. Bars indicate standard deviation. Same letter indicates no significant difference (*p* > 0.05).

O.D. measurements for the FAK ELISA at 24 h and 48 h are shown in Figure 5. At 24 h, the control showed statistically higher (*p* < 0.05) O.D. than all other groups, indicating higher levels of FAK protein phosphorylation at Y397. Groups C, C+M and C+M+F show no statistical differences (*p* > 0.05) at 24 h. At 48 h, no groups show statistical differences (*p* > 0.05), with all measurements being statistically lower (*p* < 0.05) than all 24-h measurements.

### 3.4. Mean Cell Count and Cellular Morphology

**Figure 6:**
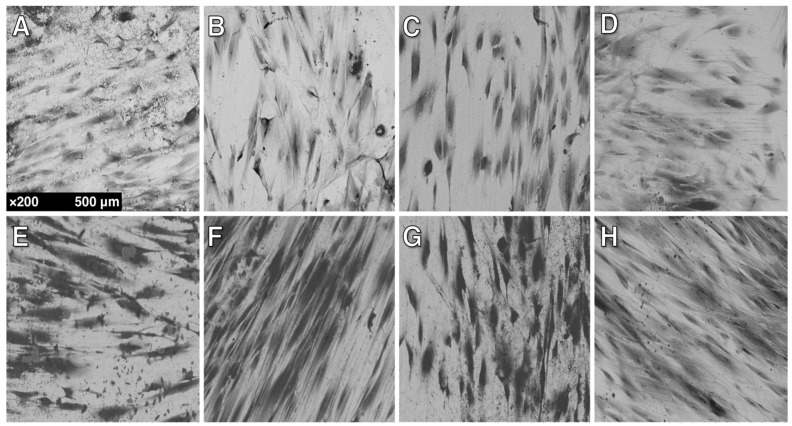
Representative SEM micrographs of HGFs on Zr surfaces at ×200: (**A**) Control, 24 h incubation; (**B**) Group C, 24 h; (**C**) Group C+M, 24 h; (**D**) Group C+M+F, 24 h; (**E**) Control, 48 h; (**F**) Group C, 48 h; (**G**) Group C+M, 48 h; (**H**) Group C+M+F, 48 h.

**Figure 7:**
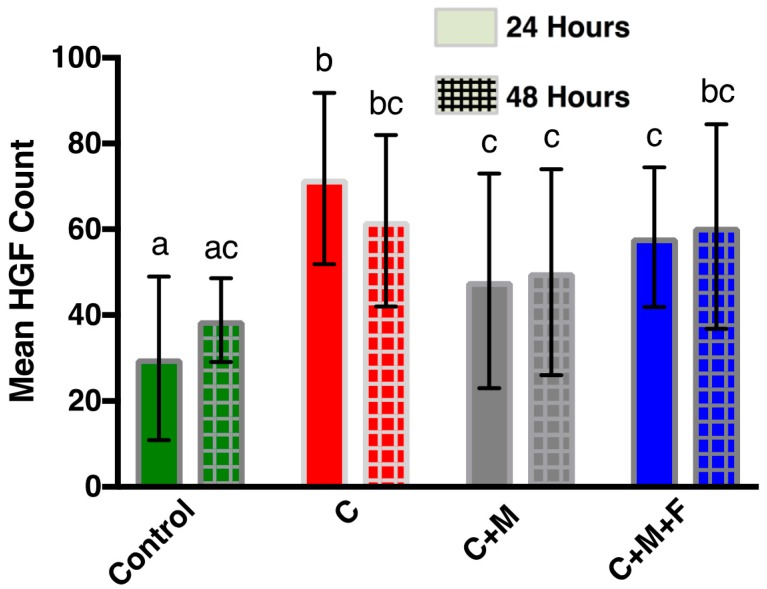
Mean cell count of HGFs present on Zr following 24 h (solid color) and 48 h (cross-hatched). Bars indicate standard deviation. Same letter indicates no significant difference (*p* > 0.05).

SEM micrographs at ×200 were used for mean cell counting and to determine cell morphology. SEM micrographs are shown in Figure 6. Mean cell count of HGFs present on Zr following 24 h and 48 h is shown in Figure 7. At 24 h, the control showed a lower (*p* < 0.05) HGF count than all other groups at 24 h. Counts of HGFs increased with statistical significance (*p* < 0.05) in group C compared to the control, but then dropped (*p* < 0.05) in group C+M and C+M+F when compared to group C. There were no statistical differences (*p* > 0.05) between C+M and C+M+F. There were no statistical differences (*p* > 0.05) between groups at 48 h. In addition, no group showed differences (*p* > 0.05) between 24 h and 48 h.

As seen in Figure 6, cells appeared to be flat, well-spread, with cellular extensions, and forming cellular bridges. There is clear anisotropy in the elongation alignment direction. In particular, this anisotropy was present for polished specimens. Furthermore, at 48 h, as compared to 24 h, HGFs were more elongated (fusiform) and spread out. HGFs appeared to orient along the polishing grooves (contact guidance by the substrate) initially produced by C polishing, as opposed to amorphous surface features or the smaller grooves produced after C+M+F polishing.

## 4. Discussion

This study evaluated the influence of clinically relevant polishing procedures for CAD/CAM Zr abutments on the attachment and proliferation of HGFs. Of important clinical significance, this work examined surface preparation strategies that are being recommended by manufacturers of Zr-based implants and polishing heads, but have not, to date, been tested in vitro. Zr is an important material for implants in light of the prevalence (approximately 0.6% of the population) of titanium allergies [44]. In addition, with the increasing prevalence of in-office CAD/CAM, there is a concomitant increase in non-standardized polishing and abrasion of abutments between individual dental offices. Thus, it is very timely to perform studies (such as ours) that directly relate to clinical practice. These studies are foundational to determine the causes of implant success or failure, particularly where it is most important; for example, in edentulous patients. As implants have shown clear evidence of increasing quality of life, improvements to implant systems, including abutments, are of clear benefit for those preferring them for cosmetic reasons [45] and to the edentulous in some situations [46]. Therefore, studies regarding abutment surfaces, with successful cellular adhesion being paramount, serve as one critical evidence-based step before eventual wide deployment of such abutment systems in patients.

### 4.1. Zr Topography

Topographic profiling results, as seen in Figure 2 and Figure 4, clearly indicated that polishing produced Zr surfaces with a reduced R_a_, quantitatively measured at two lateral length scales by both OP and AFM, as compared to the control surface. The additional polishing steps C+M and C+M+F changed the surface topography following C, but no changes in *R*_a_ were seen following C. In C+M, this was due to a lack of changes in the overall topography from C but minimization in the height deviations between the valleys and ridges. In a similar manner, C+M+F further reduced the height deviations of these grooves but introduced some finer groove structures. Furthermore, SEM and AFM micrographs of Zr surfaces (Figure 1 and Figure 3) visually showed a different topography of the control versus the polished Zr surfaces. The control micrographs revealed a polished surface with clear periodic grooves across the surface at the 100 μm SEM lateral length scale and numerous, aperiodic pits and grooves at the 30 μm AFM lateral length scale. This was expected, given the preparation steps of the Zr disks with SiC-based sanding paper. Reassuringly, C polishing resulted in a significantly smoother surface, confirmed with *R*_a_ at both lateral length scales, where bulk removal of Zr replaced the aforementioned periodic surface with flat, wide, and sparse polishing marks. This new surface topography persisted with additional polishing steps, where only a decrease in the absolute height of any valleys and ridges occurred for C+M and C+M+F. Of note, C+M+F did introduce some finer groove patterns superimposed upon the broad features produced by C+M polishing, as clearly seen in AFM and, to a lesser extent, in SEM. However, these grooves were substantially narrower (1.47 ± 0.45 μm width) and shallower (85 ± 22.0 nm depth). Relatively large standard deviations for AFM (Figure 4) revealed the expected lateral inhomogeneity of this surface when considered at such small lateral size scales, and was expected to be substantially larger than other, more macroscale, techniques, such as OP (Figure 2). Similar trends in *R*_a_ values, including consistent *R*_a_ values for changing surface topographies due to trade-offs between larger/sparse and smaller/prolific grooves, and their standard deviation from OP and AFM has previously been shown in the force dependence of Zr surface polishing [33].

### 4.2. Cellular Adhesion

Cells adhere differently to rigid substrates depending on surface topography. There are many cellular adhesion structures that have been described, but FA is among the best-defined of these. It is composed of cytoskeletal components, structural and adapter proteins, signaling molecules, and integrins (reviewed in Mitra et al. [46]). Cells receive multiple, highly complex inputs from growth factors, extracellular matrix (ECM) molecules, and mechanical stress which are regulated by integrating proteins [47]. One of outputs of multiple signaling pathways, FAK, localizes to integrin-rich cell FA sites as a non-receptor protein tyrosine kinase and regulates cell adhesion, migration, proliferation, and survival. For example, fibroblasts from mouse FAK knockouts showed excessive focal contacts, as FAK is tunable through phosphorylation. FAs can form or disassemble as needed in response to environmental and other stimuli [48]; hence, FAK signaling has a central role in promoting cell motility. In fibroblasts, FAK also controls the G1-S phase transition and growth [49]. Cells sense internal and external applied mechanical forces via a mechanism involving the activation of FAK, which provides a structure for the dynamic disassembly of FA subcomponents and cytoskeletal building blocks [50]. When stimulated by integrin activation, FAK autophosphorylates at Y397, its main autophosphorylation site. The complex process of FAK activation and its downstream signaling has been previously reviewed [47,51]. 

HGF proliferation has been shown to increase following increases in surface roughness [52]; however, other reports have shown the opposite trend [53]. The results reported herein suggest a more complex relationship. The surface (control) highest in *R*_a_ (both AFM and OP) had the lowest mean proliferation, but the highest FAK phosphorylation (at 24 h). One likely explanation is HGFs growing on the surface with the highest *R*_a_ were still actively dividing when measured, whereas the HGFs on all other surfaces, with their lower *R*_a_, were already proliferated to the limits of the surface. This may be supported by previous work that has shown smooth surfaces increase apical migration of junctional epithelium (JE) compared to rougher surfaces [54]. Generally, cells on smoother surfaces need to spread themselves and develop a strong actin cytoskeletal network in order to mechanically stabilize themselves onto the topography of the surface, as compared to surfaces with topographical features (i.e., roughness) for stabilization [19]. Therefore, smoother surfaces may promote faster proliferation to the topographical “limit” of the surface. Another factor may be the inflammatory immune response, which is known to be modulated by dental material nanotopography [55]; indeed, we have shown that healthy HGF can make a large number of inflammation-related gene transcripts [56]. As HGF are exposed to oral bacterial products in the mouth they elaborate inflammatory cytokines both in health and in periodontal disease [57].

FAK phosphorylation (Figure 5) was shown to decrease on clinically polished Zr surfaces compared to the control at 24 h. We speculate that FAK Y397 phosphorylation increased early on through 24 h as the cells increased their migration and exploration of their environment following initial attachment. However, material surfaces bind proteins from cell culture media (particularly serum) or bodily fluids within milliseconds [58]. Cell behavior is influenced by this adsorbed layer [59]. Therefore, it is entirely possible that the unpolished control surface bound the culture medium proteins in a fashion that influenced the cells to initially preferentially increase FAK Y397 phosphorylation at 24 h. At 48 h, FAK phosphorylation was lower compared to 24 h and showed no differences between any groups, so any early advantage to the cells attaching to the control surface may have been transitory, which is in line with past research [18,60]. Also, at 48 h the cells were likely less migratory and more proliferative, which would correlate with decreased FA formation [61]. More research is needed to clarify the contribution of FA formation kinetics and quality of mucosal seal formation in vivo.

Previous research has shown that the modification of the substrate modulates FA sites [11,62]. For example, FAs have been reported to increase on smoother surfaces compared to rougher surfaces [35]. Another study suggests finely grooved surfaces are optimal for FA activation in HGFs [7]. However, work by Oates et al. [11] showed that a loss of FAK did not significantly affect HGF attachment, but did affect cellular spreading. Furthermore, Baltriukienė et al. [12] showed no correlation between an in vitro testing method for adhesion via shaking and FAK phosphorylation status. This suggests that other factors or cellular products, such as fibronectin, may also be important in establishing early mucosal seal formation [7].

A direct comparison of these results with others is difficult, as the methods utilized to obtain each surface vary from study to study. While *R*_a_ is heavily utilized in dental material research, it is limited in describing complex topographies, as notably described by Wennerberg et al. [63]. *R*_a_ values are dependent on a number of factors, including cut-offs, sampling length, filters, and technique used [64]. For example, *R*_a_ values from OP are more strongly indicative of surface waviness at the microscale whereas AFM reveals underlying nanoscale roughness. Specifically, AFM typically measures surface topography with a lateral resolution of approximately 5–10 nm while OP is approximately 1–5 μm. Therefore, rather than be compared, *R*_a_ values from different techniques can be combined for novel insight into the nature of the surface. Indeed, the need for multilevel roughness assessment has recently been noted [64].

### 4.3. Other Considerations for Zr

Previous work, and the work presented herein, suggests that other factors besides surface topography, as measured by *R*_a_, may be affecting cellular responses. For example, other groups have shown that surface energy [18], chemical composition of the surface [65], and surface features not directly quantifiable by *R*_a_ affect cellular behavior [66]. For example, hydroxyl group presence on surfaces has been shown to promote influence integrin binding and so FA formation [67]. Characteristics besides *R*_a_ are important to consider, as they have been shown to be modulated by different polishing procedures on Zr [33,68]. Further work should examine cleaning procedures for CAD/CAM abutments, as post-polishing cleaning procedures could affect parameters relevant to a successful implant and cleaning protocols for abutments are not standardized [69]. In addition, Kunzler et al. [19] notes that in vitro studies carried out for two to three days or less demonstrated initial attachment, but that those results should not be extrapolated to longer periods of time, suggesting that future work at longer time scales than 48 h is needed. 

CAM diamond milling would precede the polishing procedures investigated herein. Similar to the presented work, past research has simulated CAM milling with sand paper polishing [33,68]. As this milling affects surface topography, and milling processes affect zirconia topography differently, it may affect subsequent polishing and finishing topography, and therefore cellular behavior. However, reassuringly, the presented results reveal a striking change in surface topography following clinically viable polishing that clearly removes the effects of the simulated milling process, emphasizing clinical relevance. Furthermore, differences in cellular response due to the bulk composition of CAD/CAM zirconia has been observed [21]. It is important to note that osteoblasts generally respond to roughness differently than HGFs [19,70,71], and so surface preparation for CAD/CAM abutments should be different from those for the threaded implant itself. In addition, grinding and polishing can induce a phase transformation in Zr from tetragonal to monoclinic [72]. This change in unit cell creates an increase of volume, producing compressive stresses and microcrack nucleation. Therefore, a decrease in flexure strength and low-temperature degradation (LTD) of Zr could be expected [73,74]. Among other consequences, LTD can increase *R*_a_, which may have cellular response consequences [75]. 

Finally, bulk properties of abutment materials should be considered in addition to their surfaces. In their study employing cyclic loading on zirconia and titanium abutments Mitsias et al. [76] demonstrated that titanium had higher strength and reliability as compared to zirconia. Recent work has shown that the use of a titanium base with zirconia improves the mechanical properties of abutments and combines positive elements of both materials [77]. However, Glauser et al. [78] reported no zirconia abutment fracture over a four-year period in vivo, though the relatively short time period may not be enough to detect differences. In vitro work has shown that factors like thermocycling can affect the mechanical properties of zirconia [79]. These can be modulated by the geometric arrangement (such as thickness) of the zirconia dental appliances [80]. Therefore, depending on the clinical specifics of the case, material bulk mechanics should be considered.

## 5. Conclusions

These findings suggest that polishing methodologies for Zr abutments post-computer-aided machining can significantly affect early cellular responses associated with mucosal seal formation, namely FAK phosphorylation and cell proliferation. This study investigated a three-level polishing system (course, medium and fine) typical for clinician finishing and polishing. The Zr surfaces became smoother and more regular upon polishing, as anticipated, where the fine polishing made only minor modifications to the surface post medium polishing. Concurrently, mean cell count *increased* (no significant time dependence) with the use of the polishing system while FAK protein phosphorylation at Y397 *decreased* (decreasing further with increasing time). One likely explanation is that HGFs growing on a rougher, unpolished surface were still actively dividing when measured, whereas the HGFs on all smoother, polished surfaces had already fully proliferated. Although a long-term study investigating implant health and survival at long time scales is still necessary, the present results indicated that a polishing protocol choice can potentially influence the initial 24–48 h. This is critical to mucosal seal formation, and is anticipated to be vital to long-term implant health and survival.
